# From metaphor to metric: the entropic framework as a unifying theory of aging

**DOI:** 10.1093/lifemedi/lnag021

**Published:** 2026-05-26

**Authors:** Yu Chen, Zhiyong Mao

**Affiliations:** Shanghai Key Laboratory of Maternal Fetal Medicine, Clinical and Translational Research Center of Shanghai First Maternity and Infant Hospital, Frontier Science Center for Stem Cell Research, School of Life Sciences and Technology, Tongji University, Shanghai 200092, China; Shanghai Key Laboratory of Maternal Fetal Medicine, Clinical and Translational Research Center of Shanghai First Maternity and Infant Hospital, Frontier Science Center for Stem Cell Research, School of Life Sciences and Technology, Tongji University, Shanghai 200092, China

For decades, aging research has been fragmented. The Hallmarks of Aging catalog what deteriorates, the Information Theory of Aging identifies epigenetic information loss as a key mechanism, programmed theories emphasize intrinsic timing, and damage-based theories highlight stochastic deterioration. Each perspective captures part of the phenomenon, but few integrates the others. The field lacks a common language to describe how diverse age-related changes are connected, which are causal, and where intervention should be directed. The entropic framework proposed by Wu et al. does not simply add another hypothesis. It offers a constitutive principle: a physically grounded, mathematically expressible description of the lifespan trajectory from development to senescence. It transforms entropy from a rhetorical analogy into an operational concept in aging biology.

## Beyond information theory: from molecular focus to multiscale order

The Information Theory of Aging has been influential by proposing that loss of epigenetic information drives aging [1, 2]. This insight motivated experiments such as partial reprogramming and epigenetic clock reversal. However, its explanatory scope is limited by its exclusive focus on the epigenome. Phenomena including mitochondrial cristae disorganization, lysosomal lipofuscin accumulation, extracellular matrix stiffening, and ectopic fat deposition are not easily explained as direct consequences of epigenetic change [3]. Retracing them to epigenetic origins is possible but risks post-hoc interpretation.

Wu et al. generalize the concept of information [4]. They define it in Boltzmann terms: any reduction in the number of accessible microstates under a given macrostate. This allows entropy to be quantified across biological scales: not only as CpG methylation entropy, but also as transcriptional entropy, proteostatic entropy, metabolic entropy, structural entropy, tissue organizational entropy, and systemic functional entropy.

The two frameworks are not rivals but operate at different levels. Sinclair’s theory explains a specific molecular pathway; Wu’s framework describes how order deteriorates across scales and why local perturbations propagate. Epigenetic entropy becomes one of many interdependent entropic measures. This reframing does not diminish the importance of epigenetic interventions; it clarifies that resetting epigenetic information reduces entropy not only locally but also across levels.

## From metaphor to metric: operationalizing entropy

Previous uses of entropy in aging research were largely metaphorical. Terms such as “genomic entropy” or “functional entropy” described that disorder increases [5, 6], but provided no method for calculation, no basis for cross-scale comparison, and no way to distinguish entropy-driven processes from simple damage accumulation.

Wu et al. achieve three operational advances.

First, they define each entropy metric in explicit information-theoretic terms. Methylation entropy is the Shannon entropy of binary methylation states across CpG sites. Transcriptional entropy is calculated as the deviation from a reference expression manifold. Organizational structural entropy is derived from spatial transcriptomic cell-type distributions. These definitions are computational recipes applicable to existing datasets. Standardizing these calculations across highly heterogeneous transcriptomic datasets and disparate sequencing platforms remains a significant technical barrier before the Multiscale Entropic Aging Index (MEAI) can be widely adopted.

Second, they address the non-monotonicity problem. If entropy decreases during development and increases during aging, the same numerical value can correspond to two different biological ages, making absolute entropy unusable as an aging measure. Wu et al. resolve this by measuring relative deviation from a young adult homeostatic baseline. The MEAI is defined as a *Z*-score normalized to a healthy reference population at the entropy nadir. This converts entropy from an ambiguous state variable into a directional measure of aging progression.

Third, they acknowledge the framework’s current limitations. The MEAI is presented as a conceptual scaffold, not a finished product. Unknown cross-scale weights, lack of standardized computational pipelines, the distributional dependence of Shannon entropy, and the potential utility of complementary measures such as Mahalanobis distance are explicitly noted. This recognition of uncertainty signals a transition from dogmatic assertion to hypothesis-driven refinement.

## The three-stage model: reconciling program and stochasticity

A long-standing division in aging research opposes programmed and stochastic theories. Wu et al. dissolve this opposition by assigning each process to a distinct temporal phase.

Stage I is programmed entropy reduction. Development actively builds order, establishing DNA methylation landscapes, chromatin architecture, and tissue organization. This is information writing, not merely damage avoidance. It is indisputably programmatic.

Stage II is dynamic homeostasis. During reproductive adulthood, entropy production and dissipation are approximately balanced. This equilibrium is maintained by genetically regulated programs, such as DNA repair, autophagy, and immune surveillance, that are optimized to preserve function through the reproductive window. Their eventual decline is not accidental failure but evolved prioritization.

Stage III is systemic entropy increase. Once the force of natural selection weakens, maintenance programs are downregulated and stochastic entropy accumulation escapes containment. This stage is thermodynamically entropic: unregulated, multifactorial, and non-directional.

Programmed aging is not discarded. The rate of entropy accumulation in Stage III is influenced by the initial conditions established in Stage I and the maintenance capacity retained in Stage II, both of which are under genetic control. Programmed aging survives as a modulator of entropic dynamics, not as an autonomous suicide program.

This model reframes comparative biology. The observed differences in mammalian longevity (e.g. shrews: 3 years, humans: 80 years, bowhead whales: 200 years) can be viewed as variation in the duration and robustness of the homeostatic plateau [7]. Evolution does not select for slow aging directly; it selects for prolonged reproductive competence. Entropy accumulation is the physical cost of that prolongation. The entropic framework thus links evolutionary life-history theory with molecular geroscience.

## Multiscale propagation and criticality: toward predictive dynamics

The framework’s most empirically consequential prediction is the criticality hypothesis: entropy accumulation is not linear but reaches a tipping point beyond which systemic collapse becomes inevitable.

This hypothesis follows from the model’s multiscale architecture. Molecular entropy accumulates first, largely invisibly. Cellular regulatory networks buffer this disorder. When molecular noise exceeds a threshold, it manifests as cellular entropy, such as loss of cell identity, senescence-associated secretory phenotypes, and organelle dysfunction. Cellular entropy propagates to tissue entropy, including disrupted spatial organization, fibrosis, ectopic adipocytes, and immune infiltration. Tissue entropy aggregates into systemic entropy, characterized by neuroendocrine dysregulation, loss of resilience, and multimorbidity.

The criticality hypothesis generates testable predictions:

1. Entropy should accelerate before functional decline, showing a characteristic inflection point.
2. This inflection should be scale-asynchronous: molecular first, then cellular, then tissue, then systemic. The lags define windows for early intervention.
3. Interventions that extend health span should shift the critical point or flatten the acceleration curve, providing a common quantitative endpoint for evaluating diverse strategies.

Emerging evidence is consistent with this picture. Methylation entropy distinguishes long-lived individuals from age-matched controls [8]. Single-cell transcriptional entropy identifies pre-malignant states before conventional markers [9]. Electrocardiogram-based entropy predicts fracture and mortality [10]. These observations do not yet prove criticality but they exemplify the kind of data the framework is designed to organize and interpret. Notably, a key challenge lies in validating these non-linear, scale-asynchronous inflection points *in vivo*. Dense longitudinal sampling across multiple biological scales in aging models requires substantial time and resources, presenting a significant practical hurdle for the field’s transition to predictive dynamic modeling.

## Toward a physics of aging

Wu et al. aim to move aging research from a descriptive biological discipline toward a predictive physical science. They distinguish among Clausius entropy, Boltzmann entropy, and Shannon entropy, and map each to distinct biological observables. They propose a state function, the MEAI, while acknowledging its preliminary nature, analogous to early epigenetic clocks. They discuss complementary metrics and call for standardized protocols, reference cohorts, and machine-learning integration.

This is the discourse of a field building instrumentation, not merely accumulating facts. Whether the entropic framework is ultimately validated or superseded, it has already provided aging research with a constitutive equation to test, refine, and debate.

## Conclusion

For eighty years, entropy in aging biology was a metaphor without a method, a philosophy without a formula. Wu et al. have supplied both. They demonstrate that entropy can be measured, integrated across scales, and used to generate testable predictions about the trajectory of biological order over the lifespan. They reconcile program and stochasticity, molecular mechanism and systemic collapse, biophysical principle and clinical translation.

The framework is not complete. The weights of the MEAI require empirical determination. The criticality hypothesis demands rigorous longitudinal testing. Personalized entropy assessment calls for computational innovation. But the framework is now in place. The map has been drawn. Researchers can locate themselves on the entropic landscape of aging—and begin to navigate.

## References

[1] Yang J-H , Hayano M, Griffin PT et al Loss of epigenetic information as a cause of mammalian aging. Cell 2023;186:305–26.e327. 36638792 10.1016/j.cell.2022.12.027 PMC10166133

[2] Lu YR , Tian X, Sinclair DA. The information theory of aging. Nat Aging 2023;3:1486–99. 38102202 10.1038/s43587-023-00527-6

[3] Wu Z , Qu J, Zhang W et al; Aging Biomarker Consortium. Biomarkers of ageing of humans and non-human primates. Nat Rev Mol Cell Biol 2025;26:826–47. 40935898 10.1038/s41580-025-00883-8

[4] Wu Z , Wang S, Song M et al The entropic view of aging: from thermodynamics to biology. Life Med 2026;5:lnag020. 10.1093/lifemedi/lnag020 PMC13319325 42388853

[5] Riggs JE. Aging, increasing genomic entropy, and neurodegenerative disease. Neurol Clin 1998;16:757–70. 9666049 10.1016/s0733-8619(05)70093-1

[6] Yao Y , Lu WL, Xu B et al The increase of the functional entropy of the human brain with age. Sci Rep 2013;3:2853. 24103922 10.1038/srep02853 PMC3793229

[7] Tyshkovskiy A , Ma S, Shindyapina AV et al Distinct longevity mechanisms across and within species and their association with aging. Cell 2023;186:2929–49.e20. 37269831 10.1016/j.cell.2023.05.002 PMC11192172

[8] Wang HT , Xiao FH, Gao ZL et al Methylation entropy landscape of Chinese long-lived individuals reveals lower epigenetic noise related to human healthy aging. Aging Cell 2024;23:e14163. 38566438 10.1111/acel.14163 PMC11258444

[9] Bai H , Liu X, Lin M et al Progressive senescence programs induce intrinsic vulnerability to aging-related female breast cancer. Nat Commun 2024;15:5154. 38886378 10.1038/s41467-024-49106-2 PMC11183265

[10] Hong N , Cho SW, Kim J et al Entropy, assessed by homeostatic dysregulation on electrocardiograms predicts fracture and mortality. Aging Cell 2025;24:e70227. 40931490 10.1111/acel.70227 PMC12686576

